# Rhamnetin Prevents Bradykinin-Induced Expression of Matrix Metalloproteinase-9 in Rat Brain Astrocytes by Suppressing Protein Kinase-Dependent AP-1 Activation

**DOI:** 10.3390/biomedicines11123198

**Published:** 2023-12-01

**Authors:** Chuen-Mao Yang, I-Ta Lee, Li-Der Hsiao, Zih-Yao Yu, Chien-Chung Yang

**Affiliations:** 1 Graduate Institute of Biomedical and Pharmaceutical Science, Fu Jen Catholic University, New Taipei City 242062, Taiwan; chuenmao@mail.cmu.edu.tw (C.-M.Y.); lidesiao@livemail.tw (L.-D.H.); jimmy840427@gmail.com (Z.-Y.Y.); 2 School of Dentistry, College of Oral Medicine, Taipei Medical University, Taipei 110301, Taiwan; itlee0128@tmu.edu.tw; 3 Department of Traditional Chinese Medicine, Chang Gung Memorial Hospital at Taoyuan, Taoyuan 333008, Taiwan; 4 School of Traditional Chinese Medicine, College of Medicine, Chang Gung University, Taoyuan 333323, Taiwan

**Keywords:** neuroinflammation, rhamnetin, bradykinin, protein tyrosine kinases, matrix metalloproteinase-9, astrocytes

## Abstract

Bradykinin (BK) has been recognized as a stimulant for matrix metalloproteinase (MMP)-9 expression, contributing to neuroinflammation. Modulating the BK/MMP-9 pathway offers potential in the treatment of neuroinflammatory disorders. Rhamnetin (RNT), a flavonoid compound known for its antioxidant and anti-inflammatory effects, has shown promise. However, the specific mechanisms through which RNT inhibits BK-induced MMP-9 expression remain unclear. Therefore, this study aims to delve into the intricate mechanisms underlying this process. Here, we initially demonstrated that RNT effectively attenuated BK-induced MMP-9 expression and its associated cell migration in rat brain astrocyte-1 (RBA-1) cells. Further investigation revealed that BK-driven MMP-9 protein, mRNA, and promoter activity linked to cell migration relied on c-Src, Pyk2, EGFR, PDGFR, PI3K/Akt, JNK1/2, and c-Jun. This was validated by the inhibition of these effects through specific inhibitors, a finding substantiated by the introduction of siRNAs targeting these signaling molecules. Notably, the phosphorylated levels of these signaling components induced by BK were significantly reduced by their respective inhibitors and RNT, underscoring the inhibitory role of RNT in this process. These findings indicate that, in RBA-1 cells, RNT diminishes the heightened induction of MMP-9 triggered by BK through the inhibition of c-Src/Pyk2/PDGFR and EGFR/PI3K/Akt/JNK1/2-dependent AP-1 activation. This suggests that RNT holds promise as a potential therapeutic approach for addressing neuroinflammation in the brain.

## 1. Introduction

Bradykinin (BK), a pivotal peptide in the kinin family, has emerged as a significant pathogenic factor in various neuroinflammatory disorders [1,2,3,4]. Its concentration markedly rises in these brain disorders, where BK acts as a pro-inflammatory mediator, provoking the up-regulation of several inflammatory proteins in diverse types of neuronal injuries [5]. Previous investigations have demonstrated that BK induces the up-regulation of matrix metalloproteinase-9 (MMP-9) and reactive oxygen species (ROS) [6,7,8,9]. Furthermore, our comprehensive research has illuminated the intricate interplay between BK-induced elevation of pro-inflammatory mediators, such as ROS and MMP-9 in astrocytes, and the subsequent cascade leading to neuronal apoptosis [8]. This intricate relationship underscores the pivotal role of BK in orchestrating a multifaceted molecular response within the neural milieu, thereby contributing to the complex pathogenesis of neuroinflammatory disorders. As we delve deeper into unraveling the molecular mechanisms, our focus extends beyond individual components, encompassing the dynamic interactions and feedback loops that govern the progression of neuroinflammation. Elucidating these complex networks holds promise for identifying novel therapeutic targets and interventions aimed at modulating the deleterious effects associated with BK-induced neuroinflammation. Our ongoing investigations strive to expand the breadth of knowledge surrounding the involvement of BK in neuroinflammatory processes, paving the way for innovative approaches to mitigate the impact of these debilitating disorders on neuronal health and function.

MMP-9, a member of the calcium- and zinc-dependent endopeptidases, serves as a pro-inflammatory mediator intricately involved in the pathogenic processes of the nervous system. It orchestrates inflammation by regulating cellular responses, primarily degrading the extracellular matrix (ECM). This degradation leads to the chemotaxis of inflammatory cells and the breakdown of the blood–brain barrier (BBB), signifying its pivotal role in central nervous system (CNS) injuries and diseases, including acute ischemic stroke [10], Alzheimer’s disease [11], and cancer metastasis [12]. Moreover, the dynamic involvement of MMP-9 extends beyond its matrix-degrading capabilities, as it actively influences various cellular signaling pathways, contributing to the amplification of the inflammatory responses. The intricate interplay between MMP-9 and key molecular players in neuroinflammation underscores its significance as a regulatory hub in the complex network of cellular interactions within the CNS. Understanding these multifaceted roles further illuminates the potential therapeutic avenues for targeting MMP-9 to mitigate the detrimental effects associated with neuroinflammatory conditions. Consequently, the exploration of compounds capable of inhibiting the BK and MMP-9 axis holds great promise for expanding our repertoire of therapeutic strategies. This innovative approach not only seeks to disrupt the deleterious effects of BK-induced neuroinflammation but also aims to specifically target MMP-9, mitigating its impact on the ECM and the BBB. By unraveling the nuances of this molecular axis, researchers can pave the way for the development of precise and effective interventions, ushering in a new era in the treatment of brain neuroinflammatory diseases.

Flavonoids, a class of polyphenolic compounds renowned for preventing inflammatory reactions, have demonstrated their efficacy in down-regulating MMP-9 expression in inflammatory diseases [13,14,15,16,17]. Moreover, flavonoids exhibit anti-oxidative actions during inflammation, further underscoring their potential in managing neuroinflammation. In this study, our focus shifted towards rhamnetin (RNT), a specific flavonoid, to probe its potential as an anti-inflammatory agent by inhibiting BK-induced MMP-9 induction. A substantial body of literature supports the anti-inflammatory effects of RNT and its derivatives, attributing these properties to their ability to target MMPs, mitigate oxidative stress, and regulate protein kinase activities, such as MAPKs and transcription factors [18,19,20,21]. Notably, RNT has demonstrated effectiveness in various inflammatory diseases, including cancer, ischemia, and traumatic brain injury [22,23,24]. Expanding on our investigation, we aim to unravel the molecular mechanisms underlying RNT’s ability to modulate the BK/MMP-9 axis. By elucidating the specific pathways influenced by RNT, we seek to provide a more comprehensive understanding of its anti-inflammatory properties and potential therapeutic applications in the context of neuroinflammatory disorders. Furthermore, the exploration of RNT’s impact on other aspects of neuroinflammation, such as its role in mitigating oxidative stress and regulating protein kinase activities, adds a layer of complexity to our research. This multifaceted approach allows us to not only target MMP-9 but also address broader inflammatory cascades, potentially offering a more holistic and effective strategy for managing neuroinflammation. As we delve deeper into the intricate interactions between RNT and neuroinflammatory processes, our findings may contribute not only to the understanding of flavonoid-based interventions but also to the development of targeted therapies that hold promise for alleviating the impact of neuroinflammatory diseases on the CNS.

In summary, our research aims to elucidate the molecular complexity of BK in neuroinflammatory diseases, with a particular focus on its induction of MMP-9 and cell migration in rat brain astrocyte-1 (RBA-1) cells. We strive to understand the dynamic interactions between BK and these inflammatory mediators, emphasizing the intricate nature of this relationship. Considering its therapeutic potential, our study explores RNT, a well-known anti-inflammatory flavonoid. Our primary objective is to assess whether RNT can effectively inhibit BK-induced MMP-9 expression, thus delving deeper into its potential and mechanism for treating neuroinflammatory diseases. By dissecting specific pathways influenced by RNT and their impact on broader inflammatory cascades, our goal is to enhance understanding of flavonoid interventions and develop targeted therapies for neuroinflammatory diseases. This exploration marks a crucial step in advancing innovative strategies for the management and treatment of neuroinflammation.

## 2. Materials and Methods

### 2.1. Materials

Fetal bovine serum (FBS), Dulbecco’s modified Eagle’s medium (DMEM)/Ham’s nutrient mixture F-12 (F-12), and siRNAs targeting JNK1 (RSS331962, EU_253566), Akt (RSS301983, NM_033230), c-Jun (RSS240570, NM_021835), PDGFR (RSS351966, NM_031525), and c-Src (Csk-RSS321555, NM_031997) were procured from Invitrogen (Carlsbad, CA, USA). Antibodies against phospho-PDGFRβ (Tyr751, #3161), phospho-Pyk2 (Tyr402, #3291), phospho-Akt (Ser473, #9271), phospho-c-Jun (Ser63, #2361), phospho-JNK1/2 (Thr183/Tyr185, #4668), and phospho-EGFR (Tyr1173, #4407) were obtained from Cell Signaling (Danvers, MA, USA). Anti-β-actin (C4) (sc-47778), anti-phospho-c-Src (Tyr139, sc-12928-R), anti-c-Src (sc-8056), anti-PDGFRβ (sc-374573), anti-Akt (sc-8312), anti-JNK1/2 (sc-7345), and anti-c-Jun (sc-44) antibodies, as well as dasatinib (DSTN), were purchased from Santa Cruz Biotechnology (Santa Cruz, CA, USA). Anti-EGFR (ab32077) and anti-Pyk2 (ab32448) antibodies were procured from Abcam (Cambridge, UK). Anti-glyceraldehyde-3-phosphate dehydrogenase (GAPDH) (#MCA-ID4) was sourced from EnCor (Gainesville, FL, USA). Hybond C membrane and the enhanced chemiluminescence (ECL) detection system were obtained from GE Healthcare Biosciences (Buckinghamshire, UK). PF431396 was purchased from Tocris Bioscience (Bristol, UK). AG1296 and AG1478 were obtained from Biomol (Plymouth Meeting, PA, USA). BK, Pyk2 siRNA (SASI_Rn01_00044067, NM_017318), EGFR siRNA (Rn01_00081670, NM_031507), enzymes, gelatin, TRIzol, and other chemicals were obtained from Sigma (St. Louis, MO, USA). Rhamnetin, LY294002, and SP600126 were sourced from Cayman Chemicals (Ann Arbor, MI, USA). Akt inhibitor VIII (Akti VIII) was procured from Merck Millipore (Billerica, MA, USA). Tanshinone IIA (Tan IIA) was obtained from Enzo Life Sciences (Farmingdale, NY, USA).

### 2.2. Cell Culture

The RBA-1 cell line was generously provided by Professor T.C. Jou from National Yang Ming Chiao Tung University (Taipei, Taiwan) [25]. The usage of this cell line was authorized by the Chang Gung University Institutional Animal Care and Use Committee (IACUC Approval No.: CGU16-081). Primary astrocyte cultures were verified to have a purity exceeding 95%, as indicated by positive staining with anti-glial fibrillary acidic protein. Cells from passages 4 to 35 were utilized in the experiments.

### 2.3. Western Blot

Cells were rinsed with ice-cold PBS and treated with SDS-loading buffer. The denatured proteins were heated at 95 °C for 10 min, separated via SDS-PAGE, and transferred onto Hybond-C membranes. After incubating the membranes at room temperature in 5% bovine serum albumin in TTBS for 1 h, protein levels were detected using primary antibodies (diluted 1:1000 in TTBS), and the membranes were incubated overnight at 4 °C. After washing four times (10 min each) with TTBS, the membranes were probed with horseradish-peroxidase-conjugated secondary antibody (1:1500 dilution). After the secondary antibody incubation, thorough TTBS washing was carried out. Immunoreactive bands were visualized using ECL reagents and captured with a UVP BioSpectrum 500 Imaging System (Upland, CA, USA). Image densitometry analysis utilized UN-SCAN-IT gel Version 7.1 software (Orem, UT, USA).

### 2.4. Gelatin Zymography

Culture media were collected and centrifuged at 1000× *g* for 10 min at 4 °C, as previously outlined [26]. Subsequently, under non-reducing conditions, samples were electrophoretically separated on 10% SDS-PAGE copolymerized with 1 mg/mL gelatin. Gelatinase standards (a mix of human MMP-2 and MMP-9 from Chemicon, Temecula, CA, USA) were employed. SDS was removed by washing the gels twice with 2.5% Triton X-100, followed by incubation with a developing buffer at 37 °C for 72 h. The gels were then stained with 0.5% *w*/*v* Coomassie Blue R-250 for 1 h and destained to reveal gelatinolytic bands (MMP-9/2) against a dark blue background.

### 2.5. Real-Time PCR Analysis

Total RNA extraction was performed using TRIzol reagent. Subsequently, mRNA was reverse-transcribed into cDNA, followed by real-time PCR analysis using SYBR Green PCR reagents (Applied Biosystems, Branchburg, NJ, USA) and specific primers for rat MMP-9 and GAPDH mRNAs. MMP-9 mRNA levels were quantified by normalizing to GAPDH expression.

### 2.6. Transient siRNA Transfection

RBA-1 cells, grown to 70% confluence in 6-well plates, were washed with PBS and then incubated with 1 mL/well of 5% FBS-containing DMEM/F-12 before siRNA transfection. SiRNA complexes (50 nM final concentration) were transiently transfected using Genmute transfection reagent (SignaGen Laboratories, Rockville, MD, USA) and incubated at 37 °C for 5 h. Following transfection, cells were treated with fresh 5% FBS DMEM/F-12 for an additional 8 h and then maintained in serum-free DMEM/F-12 for 16 h before exposure to BK.

### 2.7. Promoter Assay

The MMP-9-luc plasmid was generated by inserting the upstream region of the rat MMP-9 promoter (−1280 to +108) into pGL3-basic vectors (Promega, Madison, WI, USA), which include the luciferase reporter system [27]. For the MMP-9 luciferase promoter assay procedure, please refer to the previously published reference [27]. Firefly luciferase activities were subsequently normalized to β-galactosidase (β-gal) activity.

### 2.8. Cell Migration Assay

Following established protocols [28], RBA-1 cells were grown to full confluence in 6-well culture plates and then underwent a 24 h serum-free medium starvation period. A central scratch was introduced in the cell monolayer using a pipette tip. Before exposure to BK, cells were pretreated with inhibitors for 1 h. Migratory cell images were recorded using a digital camera attached to an light microscope (BX51, Olympus Optics, Shinjuku, Tokyo, Japan) in three independent experiments, and data were analyzed and summarized accordingly.

### 2.9. Statistical Analysis

The results are presented as mean ± SEM and were analyzed using the GraphPad Prism version 5 Program (GraphPad, San Diego, CA, USA). The data represent the mean ± SEM of a minimum of three independent experiments. Statistical differences between individual groups were assessed through one-way ANOVA, followed by Tukey’s post hoc test. A significance level of *p* < 0.05 was utilized to determine statistical significance.

## 3. Results

### 3.1. RNT Suppresses BK-Induced Migration and MMP-9 Up-Regulation

**Figure 1:**
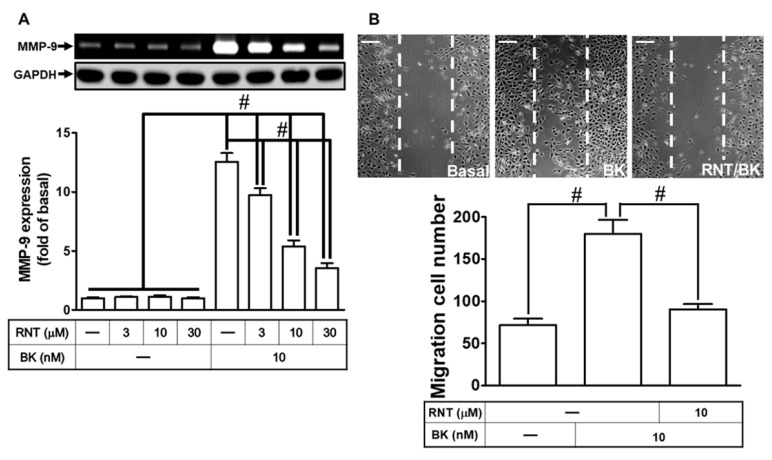
RNT suppresses BK-induced MMP-9 expression and cell migration in RBA-1 cells. (**A**) RBA-1 cells were treated with varying concentrations of RNT (3, 10, or 30 μM) for 1 h, followed by stimulation with BK (10 nM) for 24 h. Gelatin zymography assessed MMP-9 levels in media, while Western blot analyzed GAPDH as an internal control in cell lysates. (**B**) Cells were pretreated with or without RNT (10 μM) for 1 h and then exposed to BK (10 nM) for 48 h. The migratory cell count was determined. Data represent the mean ± SEM of at least three independent experiments. # *p* < 0.05 compared with cells treated with vehicle or BK, as indicated. Scale bar 200 μm.

Considering MMP-9’s implication in various brain disorders [29,30], we explored RNT’s potential neuroprotective effects against brain inflammation by targeting oxidative stress and MMPs [21]. To investigate RNT’s ability to prevent BK-induced MMP-9 up-regulation, we conducted experiments. Initially, RNT dose-dependently suppressed MMP-9 expression in RBA-1 cells treated with 10 nM BK (Figure 1A). Subsequently, MMP-9 activity was assessed using a cell migration assay. Our results revealed that 10 nM BK stimulation induced RBA-1 cell migration. Notably, pretreatment with 10 μM RNT significantly inhibited BK-induced cell migration (Figure 1B). These findings suggest that RNT possesses inhibitory effects on BK-induced MMP-9 up-regulation, correlating with the suppression of RBA-1 cell migration.

### 3.2. RNT Suppresses BK-Induced MMP-9 Expression by Inhibiting Pyk2 and c-Src Activation

**Figure 2:**
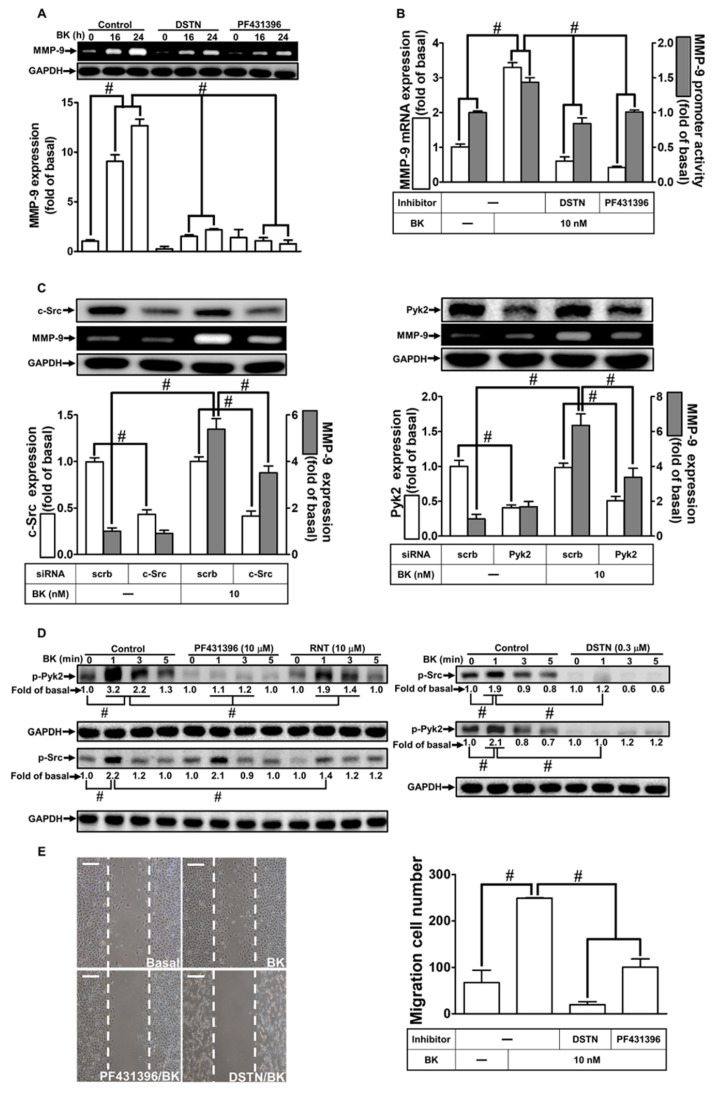
RNT suppresses BK-induced MMP-9 expression and cell migration via c-Src and Pyk2 inhibition. (**A**) Cells were pretreated with 0.3 μM DSTN or 10 μM PF431396 for 1 h, followed by stimulation with BK (10 nM) for 16 h and 24 h. MMP-9 levels were assessed by gelatin zymography. (**B**) Cells were pretreated with 0.3 μM DSTN or 10 μM PF431396 for 1 h and then incubated with BK (10 nM) for 6 h. MMP-9 mRNA levels and promoter activity were determined. (**C**) Cells were transfected with scrambled (Scrb), c-Src, or Pyk2 siRNA and then exposed to BK (10 nM) for 24 h. MMP-9 levels were analyzed by gelatin zymography, and GAPDH, c-Src, and Pyk2 levels were assessed by Western blot. (**D**) Cells were pretreated with or without 0.3 μM DSTN, 10 μM PF431396, or 10 μM RNT for 1 h, followed by BK (10 nM) challenge for indicated time intervals (1, 3, and 5 min). Phosphorylated c-Src and Pyk2 levels were determined by Western blot. (**E**) Cells were pretreated with or without 0.3 μM DSTN or 10 μM PF431396 for 1 h and then exposed to BK (10 nM) for 48 h. The migratory cell count was determined. Data represent the mean ± SEM of at least three independent experiments. # *p* < 0.05 compared with cells treated with vehicle or BK, as indicated. Scale bar 200 μm.

In a previous study, it was established that Pyk2 and c-Src play crucial roles in the up-regulation of MMP-9 and inflammatory responses triggered by BK [31]. To investigate whether these protein kinases mediate BK-induced MMP-9 expression and cell migration in RBA-1 cells, and whether RNT can inhibit these processes, we employed RNT, DSTN (a c-Src inhibitor), and PF431396 (a Pyk2 inhibitor) in our experiments. As shown in Figure 2A, pretreatment with 0.3 μM DSTN or 10 μM PF431396 significantly reduced BK-induced MMP-9 expression. Moreover, pretreatment with either 0.3 μM DSTN or 10 μM PF431396 markedly attenuated both the mRNA levels and promoter activity of MMP-9 induced by BK (Figure 2B). To validate the involvement of c-Src and Pyk2 in BK-induced MMP-9 up-regulation further, c-Src or Pyk2 siRNA transfection was used to down-regulate the protein levels of c-Src or Pyk2 individually, resulting in reduced expression of MMP-9 in RBA-1 cells exposed to BK (Figure 2C). Furthermore, BK stimulation led to a time-dependent phosphorylation of c-Src, which was effectively inhibited by pretreatment with DSTN or RNT, but not with PF431396 (Figure 2D). Similarly, BK-induced phosphorylation of Pyk2 in a time-dependent manner was significantly diminished by pretreatment with DSTN, RNT, or PF431396 (Figure 2D). Notably, pretreatment with RNT effectively inhibited both c-Src and Pyk2 phosphorylation induced by BK, indicating that c-Src acts as an upstream regulator of Pyk2 in BK-induced responses. Moreover, pretreatment with DSTN or PF431396 effectively mitigated BK-stimulated cell migration (Figure 2E). These results strongly suggest that, in RBA-1 cells, BK-induced MMP-9 up-regulation and subsequent cell migration are regulated by c-Src-dependent Pyk2 activation, and this regulatory pathway is effectively suppressed by RNT.

### 3.3. RNT Suppresses BK-Induced MMP-9 Expression via Inhibition of PDGFR and EGFR Transactivation

**Figure 3:**
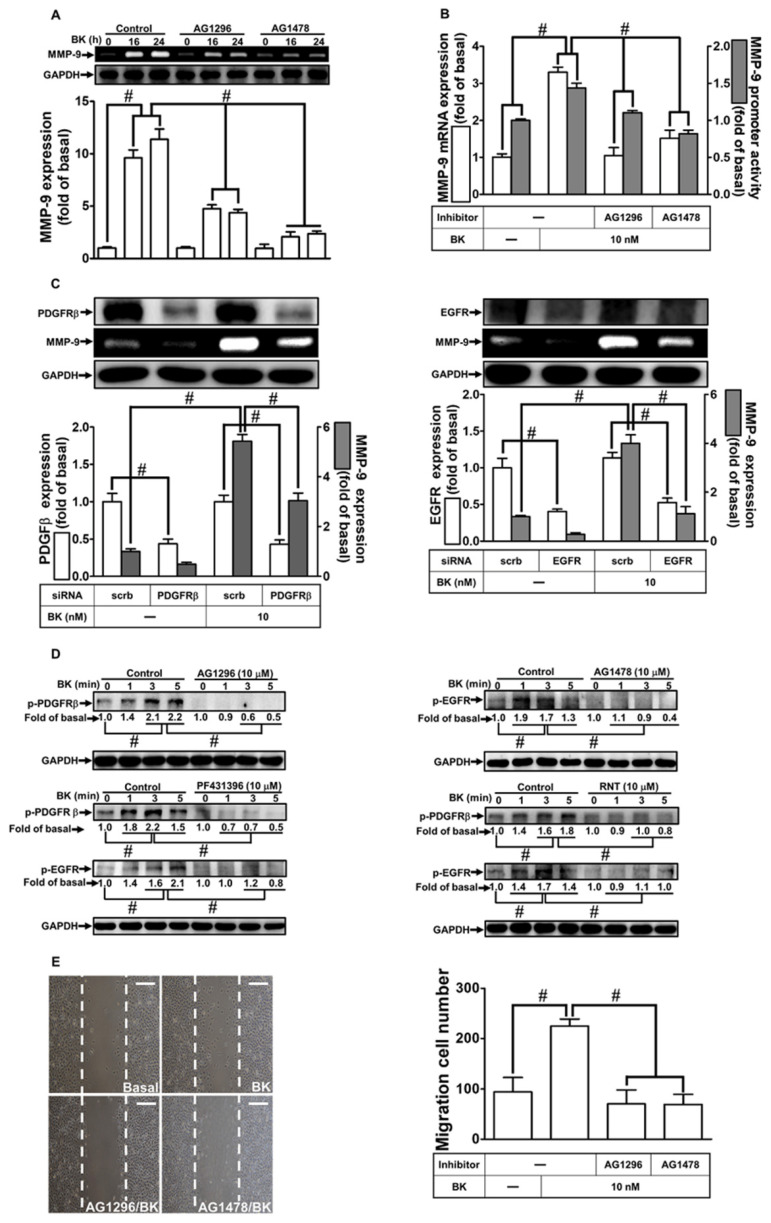
RNT suppresses BK-induced MMP-9 expression and cell migration by inhibiting PDGFR and EGFR. (**A**) Cells were pretreated with 10 μM AG1296 or 10 μM AG1478 for 1 h, followed by stimulation with BK (10 nM) for 16 and 24 h. MMP-9 levels were assessed by gelatin zymography. (**B**) Cells were pretreated with 10 μM AG1296 or 10 μM AG1478 for 1 h and then exposed to BK (10 nM) for 6 h. MMP-9 mRNA levels and promoter activity were determined. (**C**) Cells were transfected with scrambled (Scrb), PDGFR, or EGFR siRNA and then exposed to BK (10 nM) for 24 h. MMP-9 levels were analyzed by gelatin zymography, and GAPDH, PDGFR, and EGFR levels were assessed by Western blot. (**D**) Cells were pretreated with or without 10 μM PF431396, 10 μM AG1296, 10 μM AG1478, or 10 μM RNT for 1 h, followed by BK (10 nM) challenge for indicated time intervals (1, 3, and 5 min). Phosphorylated PDGFR and EGFR levels were determined by Western blot. (**E**) Cells were pretreated with or without 10 μM AG1296 or 10 μM AG1478 for 1 h and then exposed to BK (10 nM) for 48 h. The migratory cell count was determined. Data represent the mean ± SEM of at least three independent experiments. # *p* < 0.05 compared with cells treated with vehicle or BK, as indicated. Scale bar 200 μm.

Numerous studies have implicated receptor tyrosine kinases in MMP-9 up-regulation across various cell types [32,33]. Our prior research highlighted the involvement of non-receptor tyrosine kinases such as c-Src and Pyk2 in transactivating receptor tyrosine kinases to mediate MMP-9 expression [32]. Here, we investigated the role of EGFR or PDGFR engagement in BK-induced MMP-9 expression in RBA-1 cells. Using AG1296 (a PDGFR inhibitor) and AG1478 (an EGFR inhibitor), we assessed the roles of PDGFR and EGFR in BK-induced responses. Pretreatment with AG1296 or AG1478 reduced BK-stimulated MMP-9 induction (Figure 3A). Moreover, pretreating RBA-1 cells with either AG1296 or AG1478 mitigated BK-stimulated MMP-9 promoter activity and mRNA expression (Figure 3B). To confirm the roles of PDGFR and EGFR in BK-induced MMP-9 induction, cells were transfected with PDGFR or EGFR siRNA, leading to down-regulation of their respective protein expression and a reduction in MMP-9 levels induced by BK (Figure 3C). Additionally, BK stimulated the time-dependent phosphorylation of PDGFR, which was abrogated by pretreatment with AG1296, PF431396, or RNT (Figure 3D, left panel). Similarly, BK-stimulated time-dependent phosphorylation of EGFR was inhibited by pretreatment with AG1478, PF431396, or RNT (Figure 3D, right panel). These results support the fact that both EGFR and PDGFR act as downstream components of Pyk2 in BK-induced responses. Furthermore, the phosphorylation of both EGFR and PDGFR stimulated by BK was reduced by RNT. Finally, BK-induced cell migration was abrogated by pretreatment with either AG1296 or AG1478 (Figure 3E). These findings indicate that PDGFR and EGFR play roles in MMP-9 up-regulation and cell migration in RBA-1 cells exposed to BK. The inhibitory effects of RNT on BK-induced MMP-9 expression in RBA-1 cells may be mediated through blocking the phosphorylation of EGFR or PDGFR.

### 3.4. RNT Suppresses BK-Induced MMP-9 Expression and Cell Migration via Inhibition of the PI3K/Akt Pathway

**Figure 4:**
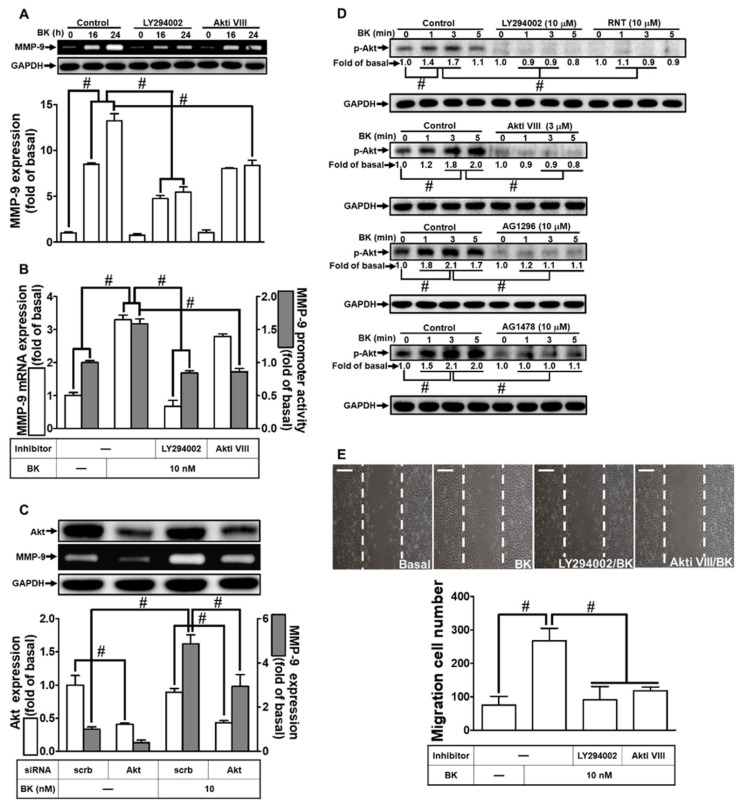
RNT suppresses BK-induced MMP-9 expression and cell migration via PI3K/Akt inhibition. (**A**) Cells were pretreated with 10 μM LY294002 or 3 μM Akti VIII for 1 h, followed by stimulation with BK (10 nM) for 16 and 24 h. MMP-9 levels were determined by gelatin zymography. (**B**) Cells were pretreated with 10 μM LY294002 or 3 μM Akti VIII for 1 h and then exposed to BK (10 nM) for 6 h. MMP-9 mRNA levels and promoter activity were determined. (**C**) Cells were transfected with scrambled (Scrb) or Akt siRNA and then exposed to BK (10 nM) for 24 h. MMP-9 levels were analyzed by gelatin zymography, and GAPDH and Akt levels were assessed by Western blot. (**D**) Cells were pretreated with or without 10 μM AG1296, 10 μM AG1478, 10 μM LY294002, 3 μM Akti VIII, or 10 μM RNT for 1 h, followed by BK (10 nM) challenge for indicated time intervals (1, 3, and 5 min). Phosphorylated Akt levels were determined. (**E**) Cells were pretreated with or without 10 μM LY294002 or 3 μM Akti VIII for 1 h and then exposed to BK (10 nM) for 48 h. The migratory cell count was determined. Data represent the mean ± SEM of at least three independent experiments. # *p* < 0.05 compared with cells treated with vehicle or BK, as indicated. Scale bar 200 μm.

A previous study has linked the PI3K/Akt signaling pathway to MMP-9 up-regulation induced by various stimuli [26]. Isorhamnetin, a derivative of RNT, has demonstrated PI3K/Akt inhibitory activity, providing protection against cardiac hypertrophy [34]. Thus, we explored whether RNT reduces BK-induced MMP-9 up-regulation by inhibiting the PI3K/Akt pathway. Using inhibitors of PI3K (LY294002) and Akt (Akti VIII), we initially dissected the roles of PI3K and Akt in BK-induced MMP-9 induction. As shown in Figure 4A, pretreatment with either LY294002 or Akti VIII significantly reduced BK-induced MMP-9 up-regulation. Similarly, LY294002 or Akti VIII pretreatment inhibited MMP-9 promoter activity and mRNA expression (Figure 4B). Furthermore, Figure 4C demonstrates the essential role of Akt in BK-stimulated MMP-9 induction, as down-regulation of Akt protein by Akt siRNA reduced MMP-9 levels induced by BK. Additionally, BK-triggered time-dependent phosphorylation of Akt was effectively inhibited by pretreatment with LY294002, Akti VIII, AG1296, AG1478, or RNT (Figure 4D). Moreover, our experiments showed that pretreatment with LY294002 or Akti VIII mitigated BK-induced RBA-1 cell migration (Figure 4E). These findings indicate the involvement of the PI3K/Akt pathway in BK-induced MMP-9 up-regulation and cell migration. Furthermore, the inhibitory effects of RNT on BK-stimulated responses in RBA-1 cells are, at least partially, mediated through suppression of the PI3K/Akt signaling pathway.

### 3.5. BK-Induced MMP-9 Up-Regulation in RBA-1 Cells Is Mediated through JNK1/2 Activation

**Figure 5:**
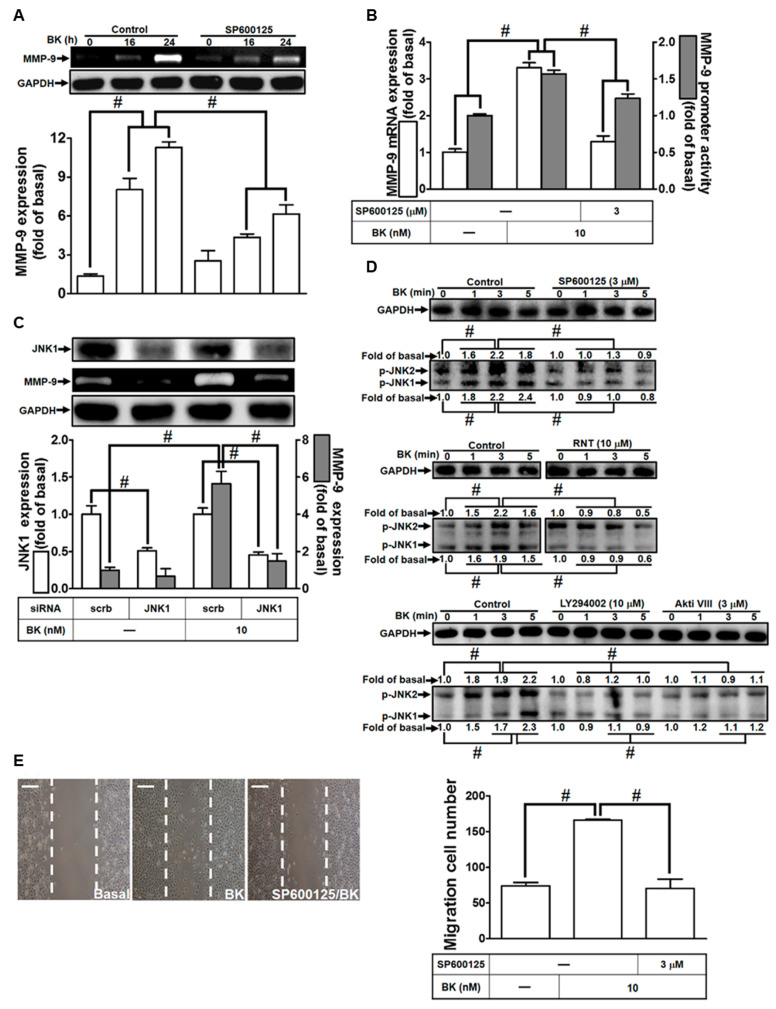
Regulation of BK-induced MMP-9 expression and cell migration by JNK1/2 signaling in RBA-1 cells. (**A**) Cells were pretreated with 3 μM SP600125 for 1 h, followed by stimulation with BK (10 nM) for 16 and 24 h. MMP-9 levels were assessed through gelatin zymography. (**B**) Cells were pretreated with 3 μM SP600125 for 1 h and then exposed to BK (10 nM) for 6 h. MMP-9 mRNA expression and promoter activity were measured. (**C**) Cells were transfected with scrambled (Scrb) or JNK1 siRNA and then exposed to BK (10 nM) for 24 h. MMP-9 levels in the media and cell lysates were determined using gelatin zymography. Additionally, GAPDH and JNK1 levels were assessed through Western blot. (**D**) Cells were pretreated with or without 3 μM SP600125, 10 μM LY294002, 3 μM Akti VIII, or 10 μM RNT for 1 h and then challenged with BK (10 nM) for indicated time intervals. Levels of JNK1/2 phosphorylation were determined by Western blot. (**E**) Cells were pretreated with or without 3 μM SP600125 for 1 h and then exposed to BK (10 nM) for 48 h. Migratory cell count was determined. Data represent the mean ± SEM of at least three independent experiments. Significant differences (# *p* < 0.05) were observed compared with cells exposed to the vehicle or BK, as indicated. Scale bar 200 μm.

The involvement of JNK1/2 signaling has been recognized in various physiological and pathological contexts across different cell types [35]. Consequently, we investigated whether RNT reduced BK-induced responses in RBA-1 cells by regulating JNK1/2 activation. Initially, we employed the JNK1/2 inhibitor SP600125 in RBA-1 cells to explore its effects on BK-up-regulated MMP-9. As depicted in Figure 5A, pretreating cells with SP600125 inhibited MMP-9 expression stimulated by BK. Furthermore, pretreatment with SP600125 reduced BK-stimulated MMP-9 promoter activity and mRNA expression (Figure 5B). We further confirmed the role of JNK1/2 in BK-induced MMP-9 expression by transfecting JNK1 siRNA, which reduced JNK1 protein production and decreased BK-provoked MMP-9 expression (Figure 5C). Additionally, phosphorylation of JNK1/2 increased upon BK exposure for the examined time intervals, which was attenuated by pretreatment with SP600125, LY294002, Akti VIII, or RNT (Figure 5D). These findings indicate that JNK1/2 acts as a downstream signaling component of the PI3K/Akt pathway. To evaluate the functional role of JNK1/2, cells were pretreated with SP600125 and then exposed to BK to observe cell migration at 48 h. The images in Figure 5E demonstrate that BK-induced cell migration is mitigated by SP600125. These results suggest that BK-induced MMP-9 expression and migration of RBA-1 cells are mediated through JNK1/2 phosphorylation, which is also inhibited by RNT.

### 3.6. RNT Suppresses BK-Stimulated MMP-9 Expression in RBA-1 Cells by Inhibiting c-Jun Activation

**Figure 6:**
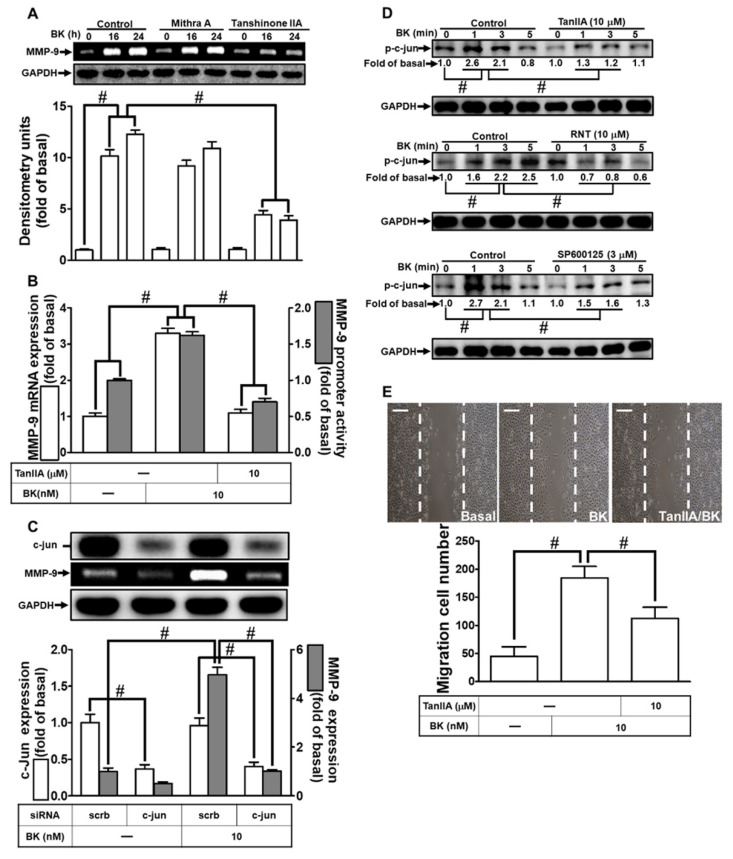
RNT suppresses BK-induced AP-1 activation, MMP-9 expression, and cell migration. (**A**) Cells were pretreated with 1 μM Mithra A or 10 μM Tan IIA for 1 h, followed by stimulation with BK (10 nM) for 16 and 24 h. MMP-9 levels were assessed by gelatin zymography. (**B**) Cells were pretreated with 10 μM Tan IIA for 1 h and then incubated with BK (10 nM) for 6 h. MMP-9 mRNA expression and promoter activity were evaluated. (**C**) Cells were transfected with scrambled (Scrb) or c-Jun siRNA and then treated with BK (10 nM) for 24 h. MMP-9 levels were determined by gelatin zymography, and GAPDH and c-Jun were analyzed by Western blot. (**D**) Cells were pretreated with 3 μM SP600125, 10 μM Tan IIA, or 10 μM RNT for 1 h and then challenged with BK (10 nM) for various time intervals. Phosphorylated c-Jun levels were determined by Western blot. (**E**) Cells were pretreated with 10 μM Tan IIA for 1 h and then exposed to BK (10 nM) for 48 h. Migratory cells were counted. Data represent the mean ± SEM of at least three independent experiments. # *p* < 0.05 compared with cells treated with vehicle or BK, as indicated. Scale bar 200 μm.

AP-1 is implicated in various brain inflammatory responses [36], and its activation by pro-inflammatory mediators can lead to MMP-9 up-regulation in diverse cell types [37,38]. Additionally, there is evidence suggesting cooperation between Sp1 and AP-1 in MMP-9 expression under various conditions [39,40]. However, the specific roles of AP-1 and Sp1 in BK-induced MMP-9 up-regulation remain unclear. To investigate the involvement of AP-1 and Sp1 in BK-stimulated MMP-9 expression, we utilized Tan IIA and Mithra A, respectively. Tan IIA significantly reduced BK-induced MMP-9 expression, while Mithra A had no effect (Figure 6A). Furthermore, Tan IIA pretreatment attenuated BK-induced MMP-9 promoter activity and mRNA levels (Figure 6B). Confirming the role of c-Jun (a subunit of AP-1) in MMP-9 expression, c-Jun siRNA transfection resulted in reduced c-Jun levels and decreased BK-induced MMP-9 expression (Figure 6C). We also explored whether phosphorylation of c-Jun was essential for BK-induced responses. BK time-dependently increased phosphorylated c-Jun levels, and pretreatment with Tan IIA, LY294002, Akti VIII, SP600125, or RNT inhibited BK-induced c-Jun phosphorylation (Figure 6D). Examining RBA-1 cell migration induced by BK to assess AP-1 function, Tan IIA pretreatment significantly blocked BK-stimulated cell migration within 48 h (Figure 6E). These findings indicate that AP-1 activation in RBA-1 cells is regulated by the JNK1/2-dependent signaling pathway and plays a crucial role in BK-induced MMP-9 expression and cell migration. Moreover, RNT inhibits BK-induced responses, partly by suppressing AP-1 activation.

## 4. Discussion

The presence of BK has been strongly linked to inflammatory conditions within the brain. Notably, BK has been implicated in contributing to neuroinflammation by up-regulating MMP-9. In light of this, our current study seeks to unravel the intricate mechanisms that drive MMP-9 induction by BK. Additionally, our goal is to identify potential pharmaceutical agents with the capacity to target and modulate MMP-9 expression specifically in the context of neuroinflammation. In this investigation conducted on RBA-1 cells, we systematically explored the molecular pathways through which RNT inhibits BK-induced MMP-9 up-regulation. Our results indicate that RNT effectively mitigates BK-induced MMP-9 expression, concurrently impeding the migration of RBA-1 cells. Notably, these effects are, at least in part, mediated by the inhibition of the c-Src/Pyk2/PI3K/Akt/JNK1/2-dependent AP-1 activation in RBA-1 cells. The observed reduction in MMP-9 expression by RNT suggests its potential as a therapeutic intervention in neuroinflammatory conditions associated with BK. This finding aligns with the broader context of inflammatory responses in the brain and underscores the significance of targeting MMP-9 in mitigating the detrimental effects of neuroinflammation. This mechanistic understanding contributes to the rationale for considering RNT as a promising candidate for further development as a therapeutic agent for neuroinflammatory conditions linked to BK.

Beyond their roles in fundamental cellular processes, the non-receptor tyrosine kinases c-Src and Pyk2 exhibit multifaceted functions that extend into the intricate realms of neurobiology and neurological disorders. Their involvement in various cellular activities, such as proliferation, migration, survival, and adhesion, underscores their significance in orchestrating the complex interaction of events crucial for normal cell functioning [41,42]. In the context of neurobiology, these kinases play a vital role in neuronal differentiation and the intricate development of the nervous system. Their regulatory influence extends to processes that are fundamental for shaping the architecture and functionality of neural networks. The direct activation of focal adhesion kinase and Pyk2 by c-Src adds another layer of complexity, as these signaling pathways are essential for the organization of the cytoskeleton. The cytoskeleton, in turn, is a structural framework critical for maintaining cell shape, supporting intracellular transport, and facilitating cellular migration. Importantly, the implications of c-Src and Pyk2 extend beyond normal cellular processes and into the pathophysiology of neuroinflammatory and neurodegenerative disorders. The well-documented functions of these kinases in such conditions highlight their potential as key players in the intricate cascade of events leading to neuronal damage and dysfunction [43,44,45]. Our prior research has shed light on the intricate interplay between c-Src and Pyk2 within the nervous system [33]. The c-Src/Pyk2 signaling pathway, as evidenced in numerous studies, emerges as a crucial participant in the up-regulation of MMP-9 induced by various proinflammatory mediators [26,32,33]. Specifically, in a previous study, we elucidated that BK amplifies MMP-9 expression through the activation of the c-Src/Pyk2 signaling cascade [31]. While direct evidence of RNT inhibiting the c-Src/Pyk2 signaling pathway is currently lacking, noteworthy insights emerge from the chemoprotective effects of its derivative, isorhamnetin, observed in colon cancer [46]. This chemoprotective effect has been linked to the inhibition of oncogenic c-Src [46]. This intriguing parallel aligns with our current findings, where transfection with Pyk2 or c-Src siRNA resulted in the suppression of BK-stimulated MMP-9 expression. Furthermore, RNT demonstrated the ability to mitigate the phosphorylation of both Pyk2 and c-Src. In light of these observations, our results suggest that RNT exerts regulatory control over BK-induced MMP-9 expression and cell migration in RBA-1 cells by specifically inhibiting the activities of Pyk2 and c-Src. This dual inhibitory effect on key components of the c-Src/Pyk2 signaling pathway positions RNT as a potential modulator of neuroinflammatory responses and cell migratory processes. Future investigations may delve deeper into the specific molecular interactions between RNT and the c-Src/Pyk2 pathway, paving the way for a more comprehensive understanding of its therapeutic implications in neuroinflammation and related conditions.

Receptor tyrosine kinases (RTKs) stand as key regulators of essential neuronal functions, playing critical roles in processes such as cell survival, neurogenesis, and synaptogenesis. The intricate orchestration of these signaling components is paramount for maintaining neuronal health. Notably, dysregulation of RTKs has been implicated in various neurodegenerative diseases, underscoring their significance in the pathophysiology of these conditions [47]. Recent evidence has brought attention to the ability of BK to stimulate the transactivation of RTKs [48]. This newfound insight adds a layer of complexity to our understanding of BK’s role in neuronal signaling. In the context of our prior studies, we have consistently emphasized the pivotal role of RTK signaling in the up-regulation of MMP-9 under diverse challenges [32,33]. Our ongoing research endeavors aim to dissect the specific interactions between BK, RTKs, and MMP-9, thereby providing comprehensive insights into the intricate signaling cascades at play in neurodegenerative diseases. By elucidating the regulatory roles of RTKs in MMP-9 up-regulation, we aspire to contribute to the identification of potential therapeutic targets for mitigating the impact of dysregulated signaling in neurodegenerative conditions. In the course of this investigation, we have uncovered that the attenuation of PDGFR or EGFR can effectively suppress BK-induced MMP-9 expression in RBA-1 cells. This not only elucidates the involvement of PDGFR and EGFR in the induction of MMP-9 by BK in these cells but also provides a better understanding of the intricate signaling pathways at play. Furthermore, our study reveals that the phosphorylation of PDGFR and EGFR is diminished by the upstream signaling inhibitors Pyk2 and RNT. This establishes Pyk2 as a crucial upstream signaling molecule for PDGFR and EGFR in the context of BK-induced responses. These findings align seamlessly with our earlier reports, emphasizing the regulation of RTK transactivation by c-Src and Pyk2 signaling pathways, particularly in the context of MMP-9 expression induced by various stimuli [27,33]. To extend our understanding, we explored the impact of PDGFR or EGFR inhibitors on BK-induced cell migration. Notably, pretreatment with these inhibitors demonstrated a significant mitigation of BK-induced cell migration, mirroring the effects observed with RNT pretreatment. This robust evidence strongly suggests that RNT exerts its inhibitory effects on BK-induced MMP-9 up-regulation and cell migration, at least partially, by attenuating Pyk2-dependent PDGFR and EGFR transactivation in RBA-1 cells. These findings underscore the intricate interplay between Pyk2, PDGFR, and EGFR in the context of BK-induced responses, and provide a mechanistic understanding of how RNT modulates these signaling pathways. Future investigations may delve deeper into the specific molecular interactions and downstream signaling events to refine our understanding and pave the way for the development of targeted therapeutic strategies.

The intricate PI3K/Akt pathway plays a central role in inflammation, governing essential cellular processes such as survival, proliferation, differentiation, and metabolism. Upon activation, this pathway exerts a profound influence on various cellular functions, including immune and inflammatory responses. Notably, the PI3K/Akt pathway has been implicated in the up-regulation of MMPs, notably MMP-9, across diverse cell types and in response to a range of stimuli [32,33]. BK has been consistently observed to activate the PI3K/Akt pathway in multiple cell types, indicating its involvement in cellular responses [7,49]. In our previous investigation with RBA-1 cells, we provided evidence underscoring the regulatory role of PI3K/Akt signaling in BK-induced MMP-9 expression [7]. This underscores the significance of the PI3K/Akt pathway as a critical mediator in the cellular response to BK, particularly concerning MMP-9 expression. In the current study, we strategically utilized Akt siRNA transfection and an Akt inhibitor to underscore the pivotal role of PI3K/Akt signaling in BK-induced MMP-9 expression. Unswervingly, our data demonstrate that both Akt siRNA transfection and pretreatment with Akti VIII significantly reduce BK-induced MMP-9 expression in RBA-1 cells. Furthermore, the application of Akti VIII or LY294002 effectively impedes BK-induced migration of RBA-1 cells. Additionally, the phosphorylation levels of Akt are notably diminished by pretreatment with LY294002, Akti VIII, AG1498, AG1296, and RNT. These findings strongly support the notion that the PI3K/Akt pathway functions downstream of PDGFR and EGFR, playing a pivotal role in mediating BK-induced MMP-9 up-regulation and cell migration in RBA-1 cells. The inhibitory effects of RNT on BK-induced MMP-9 up-regulation and cell migration in RBA-1 cells were, at least partially, accomplished by attenuating the PI3K/Akt signaling pathway. It is noteworthy to highlight the diverse responses to RNT observed in different studies. For instance, a study by Park et al. demonstrated that rhamnetin, a derivative of RNT, induces a cytoprotective effect on H9c2 cardiomyoblasts by restoring Akt phosphorylation attenuated by H_2_O_2_ [23]. These contrasting outcomes may be attributed to differences in cell types or stimuli, underlining the context-dependent nature of cellular responses to RNT.

MAPKs, encompassing JNK1/2, ERK1/2, and p38 MAPK, represent shared downstream signaling effectors of RTKs in response to inflammatory stimuli. Particularly, JNK1/2 activation has been strongly linked to neuroinflammation [50]. It is noteworthy that BK, a key player in inflammatory responses, has been demonstrated to stimulate JNK1/2 activity, contributing to the induction of various inflammatory mediators in the brain [51]. The convergence of MAPKs as downstream effectors of RTK signaling highlights their significance in orchestrating cellular responses to inflammation. Specifically, the activation of JNK1/2, as observed in response to BK, underscores its role as a critical mediator in neuroinflammatory processes. This activation may initiate a cascade, leading to the expression of pro-inflammatory mediators, thereby contributing to the intricate network of neuroinflammatory responses. In light of these findings, our study aims to contribute to the comprehensive understanding of the signaling pathways modulated by BK in neuroinflammation. By elucidating the involvement of MAPKs, particularly JNK1/2, in BK-induced inflammatory responses, we gain valuable insights into potential targets for therapeutic interventions in neuroinflammatory disorders. The intricate interplay between BK and JNK1/2 activity provides a foundation for further investigations, where delineating the specific molecular mechanisms may unveil novel opportunities for targeted therapeutic strategies. In this study, we employed both a JNK1/2 inhibitor (SP600125) and JNK1 siRNA transfection to substantiate the role of JNK1/2 in BK-induced responses. Our results consistently demonstrated that pretreatment with SP600125 and JNK1 siRNA transfection effectively attenuated BK-induced MMP-9 expression and blocked BK-induced cell migration. Additionally, pretreatment with SP600125, LY294002, Akti VIII, or RNT significantly reduced the phosphorylation of JNK1/2 activated by BK. These findings align with a study by Jnawali et al., suggesting that RNT can suppress nitric oxide and cytokine production in lipopolysaccharide-stimulated macrophages by binding to JNK1 and p38 MAPK with high affinity [52]. The convergence of our results with this study supports the notion that BK-induced MMP-9 expression is intricately regulated by PI3K/Akt-dependent JNK1/2 activation. Our study contributes to the understanding of the inhibitory effects of RNT on BK-induced MMP-9 up-regulation and cell migration in RBA-1 cells. Notably, these effects were, at least partially, achieved by attenuating the JNK1/2 signaling pathway. This adds another layer of complexity to the regulatory mechanisms governing neuroinflammatory responses, where the crosstalk between PI3K/Akt and JNK1/2 pathways becomes a critical focal point.

The AP-1 protein complex, encompassing members like c-Fos, c-Jun, ATF, and JDP families, assumes a pivotal role in orchestrating DNA transcription, particularly in the context of inflammatory responses [53]. Among these constituents, c-Jun stands out as a common target activated by various pro-inflammatory agents [53]. It is closely associated with MMP-9 overexpression in different cell types or tissues exposed to pro-inflammatory stimuli, underscoring its significance in neurological inflammation. In this study, our investigation delved into the potential of JNK1/2-dependent signaling in RBA-1 cells to activate AP-1, thereby enhancing MMP-9 expression and cell migration induced by BK. Notably, we observed that the use of the AP-1 inhibitor Tan IIA resulted in a notable reduction in MMP-9 expression in RBA-1 cells challenged with BK. These findings were further supported by c-Jun siRNA transfection, which led to a down-regulation of MMP-9 expression in RBA-1 cells exposed to BK. Additionally, the phosphorylation of c-Jun induced by BK was effectively suppressed by pretreating cells with Tan IIA, SP600125, or RNT. These data strongly suggest that JNK1/2 serves as an upstream regulator of AP-1 in this context. Therefore, our study concludes that, in RBA-1 cells, BK-induced MMP-9 expression is modulated by PI3K/Akt-dependent JNK1/2 signaling, leading to the activation of AP-1. Furthermore, RNT exhibits inhibitory effects on AP-1 activation in RBA-1 cells, thereby suppressing BK-induced MMP-9 expression. These findings align with a study on isorhamnetin, demonstrating its anti-inflammatory effects linked to the inhibition of AP-1 activation induced by TNF-α in human umbilical vein endothelial cells [54]. Although Sp1 has been shown to cooperate with AP-1 in up-regulating MMP-9 expression in response to certain stimuli in various cell types, it does not play a role in these BK-induced responses. Overall, our results indicate that RNT inhibits BK-induced MMP-9 expression and cell migration by suppressing AP-1 transcriptional activity, providing valuable insights for potential therapeutic strategies in neuroinflammatory disorders.

**Figure 7:**
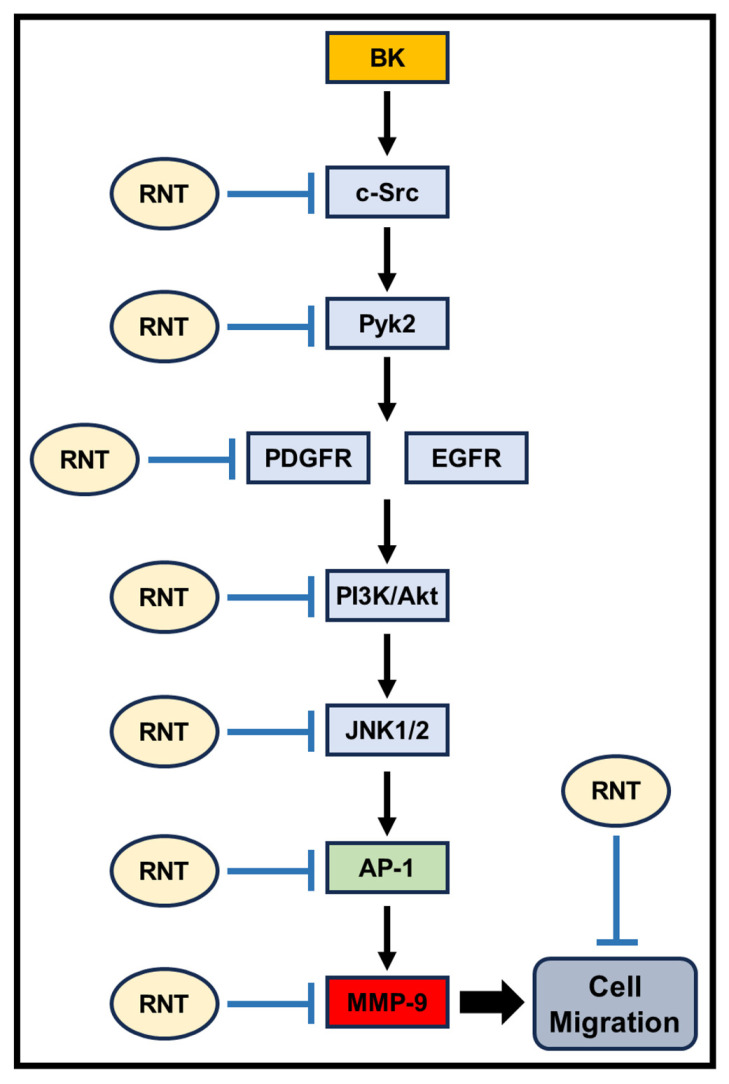
A schematic diagram is employed to depict how RNT inhibits BK-induced MMP-9 expression and cell migration in RBA-1 cells. Exposure to BK prompts an increase in MMP-9 expression and cell migration in RBA-1 cells by activating c-Jun. This activation relies on the c-Src/Pyk2/PDGFR and EGFR/PI3K/Akt/JNK1/2 signaling pathways. The inhibitory impact of RNT on BK-induced MMP-9 expression and cell migration involves suppressing the c-Src/Pyk2/PDGFR and EGFR/PI3K/Akt/JNK1/2 cascade. Additionally, RNT impedes c-Jun’s transcriptional activity. Activation is indicated by arrows (→), and inhibition is denoted by perpendicular lines (⊥).

In summary, our study reveals that BK exposure induces the up-regulation of MMP-9 and cell migration in RBA-1 cells, with intricate regulation by activated c-Jun. This activation is largely dependent on the c-Src/Pyk2/PDGFR and EGFR/PI3K/Akt/JNK1/2 signaling cascade, as depicted in Figure 7. Furthermore, our findings demonstrate that RNT inhibits signaling pathways involving c-Src/Pyk2/PDGFR, EGFR/PI3K/Akt, JNK1/2, and the transcriptional activity of c-Jun in RBA-1 cells. This comprehensive inhibition leads to reduced BK-induced MMP-9 expression and cell migration. As a result, RNT emerges as a promising candidate for treating neuroinflammation in the CNS, particularly in response to various insults.

## Data Availability

The data presented in this study are available on request from the corresponding author.
